# Supplementary material for: Prevalence and determinants of opportunistic infections among HIV-infected adults receiving antiretroviral therapy in Ethiopia: A systematic review and meta-analysis
Source: Front Med (Lausanne). 2023 Feb 16;10:1087086. doi: 10.3389/fmed.2023.1087086 (PMC9978215; doi:10.3389/fmed.2023.1087086)
Supplement: Supplementary file 1 [file Data_Sheet_1.pdf]

Quality assessment of studies using JBI's critical appraisal tools designed for Descriptive cross-sectional study

Study	Sample size	JBI's critical appraisal questions									Score	Overall Appraisal
		Q1	Q2	Q3	Q4	Q5	Q6	Q7	Q8	Q9		
Alemayehu et *al*	362	Y	Y	Y	Y	y	Y	Y	Y	Y	9	Included

Y –Yes;N-No;U -Unclear-Question. Overall score is calculated by counting the number of Y's in each row.Q1=Was the sample frame appropriate to address the target population? Q2=Were study participants sampled in an appropriate way? Q3=Was the sample size adequate? Q4=Were the study subjects and the setting described in detail? Q5=Was the data analysis conducted with sufficient coverage of the identified sample? Q6=Were valid methods used for the identification of the condition? Q7=Was the condition measured in a standard, reliable way for all participants? Q8=Was there appropriate statistical analysis? Q9=Was the response rate adequate, and if not, was the low response rate managed appropriately?

Quality assessment of studies using JBI's critical appraisal tools designed for Analytical cross-sectional study

Study	Sample size	JBI's critical appraisal questions								Score	Overall Appraisal
		Q1	Q2	Q3	Q4	Q5	Q6	Q7	Q8		
Debasu et al.[19]	360	Y	y	y	y	y	y	y	y	8	Included
Addisu et al.	315	y	y	y	y	y	y	y	y	8	Included
Wachamo et al.	414	Y	y	y	y	y	y	y	y	8	Included
Mitiku et al.	358	Y	y	y	y	y	y	y	y	8	Included
Weldearegawi et al	400	y	y	y	y	y	y	y	y	8	Included
Dereje et al.	386	Y	y	y	y	y	y	y	y	8	Included
Mengistu et al	266	Y	y	y	y	y	y	y	y	8	Included
Moges et al.	423	y	y	y	y	y	y	y	y	8	Included
Hailu et al.	394	Y	y	y	y	y	y	y	y	8	Included
Fite et *al.*	303	Y	y	y	y	y	y	y	y	8	Included
Solomon et *al.*	658	y	y	y	y	y	y	y	y	8	Included
Tiku and Tirhas	125	Y	y	y	y	y	y	y	y	8	Included

Y –Yes;N-No;U -Unclear-Question. Overall score is calculated by counting the number of Y's in

For analytical cross-sectional study, the JBI checklist assessed the following questions Q1= were the criteria for inclusion in the sample clearly defined? Q2=were the study subjects and the=Were objective, standard criteria used for measurement of the condition? Q5=Were confounding factors identified? Q6=Were strategies to deal with confounding factors stated? Q7=Were the outcomes measured in a valid and reliable way? Q8=Was appropriate statistical analysis used?

JBI Critical Appraisal Checklist for Cohort Studies

Study	Sample size	JBI's critical appraisal questions											Score	Overall Appraisal
		Q1	Q2	Q3	Q4	Q5	Q6	Q7	Q8	Q9	Q10	Q11		
Arefaine et *al*	317	Y	y	y	y	y	y	y	y	y	y	y	8	Included
Direslegn et *al.*	232	y	y	y	y	y	y	y	y	y	y	y	8	Included

- ✓ Q1 Were the two groups similar and recruited from the same population?
- ✓ Q2Were the exposures measured similarly to assign people to both exposed and unexposed groups?
- ✓ Q3 Was the exposure measured in a valid and reliable way?
- ✓ Q4Were confounding factors identified?
- ✓ Q5 Were strategies to deal with confounding factors stated?
- ✓ Q6 Were the groups/participants free of the outcome at the start of the study (or at the moment of exposure)?
- ✓ Q7Were the outcomes measured in a valid and reliable way?
- ✓ Q8 Was the follow up time reported and sufficient to be long enough for outcomes to occur?
- ✓ Q9 Was follow up complete, and if not, were the reasons to loss to follow up described and explored?
- ✓ Q 10 Were strategies to address incomplete follow up utilized?
- ✓ Q 11 Was appropriate statistical analysis used?
