# Supplementary material for: Prevalence and determinants of opportunistic infections among HIV-infected adults receiving antiretroviral therapy in Ethiopia: A systematic review and meta-analysis
Source: Front Med (Lausanne). 2023 Feb 16;10:1087086. doi: 10.3389/fmed.2023.1087086 (PMC9978215; doi:10.3389/fmed.2023.1087086)
Supplement: Supplementary file 2 [file Data_Sheet_2.pdf]

Concept map

**Concept map 1:**prevalence	Keywords	Prevalence [Text Word]
	MeSH terms	"Prevalence"[Mesh] OR "Incidence"[Mesh]
**Concept map 2:**opportunistic infections	Keywords	opportunistic infections[Text Word]
	MeSH terms	"opportunistic infections"[MeSH Terms] OR "AIDS-Related Opportunistic Infections"[Mesh]
**Concept map 3:**HIV	Keywords	HIV[Text Word]
	MeSH terms	"hiv"[MeSH Terms]
Concept map 4:risk factors	Keywords	Risk Factors[Text Word]
	MeSH terms	"Risk Factors"[Mesh]
**Concept map 5:**adults	Keywords	adults[Text Word]
	MeSH terms	"adult"[MeSH Terms]
**Concept map 6:**Ethiopia	Keywords	Ethiopia (text word)
	MeSH terms	"Ethiopia"[Mesh]

((((((((((Prevalence [Text Word]) OR ("Prevalence"[Mesh] AND (opportunistic infections[Text Word])) OR "AIDS-Related Opportunistic Infections"[Mesh])) AND (HIV[Text Word])) OR ("hiv"[MeSH Terms])) AND Predictors[Text Word] OR (Risk Factors[Text Word]))OR ("Associated factors"[Text Word] OR ("Risk Factors"[Mesh])) AND (adults[Text Word])) OR ("adult"[MeSH Terms])) "anti-retroviral agents"[MeSH Terms] OR antiretroviral [Text Word] AND (Ethiopia (text word))) OR ("Ethiopia*"[Mesh])
